# Psychoanalysis in the treatment of autism: why is France a cultural outlier?

**DOI:** 10.1192/bjb.2020.138

**Published:** 2021-04

**Authors:** D. V. M. Bishop, Joel Swendsen

**Affiliations:** 1 University of Oxford, UK; 2 National Centre for Scientific Research, France

**Keywords:** Psychoanalysis, autism, culture, child abuse, child sexuality

## Abstract

In most countries, social or behavioural interventions are recommended for autism. However, in France, psychoanalysis is still used, despite objections by patients, families and mental health experts. Supporters of psychoanalysis maintain that the choice of therapeutic approach is a matter of cultural preference, and that objections to psychoanalysis arise from misunderstandings. We argue that more deep-rooted problems are the lack of an evidence base for psychoanalysis and its focus on sexual relationships between children and adults, which demonises mothers and can put children at risk of abuse. Furthermore, psychoanalysis in France is protected from criticism by powerful educational and political networks.

## Psychoanalysis as an intervention for child language disorders

There have always been geographical differences in the practice of medicine, even within Western societies, but in psychiatry, especially child psychiatry, national variations are taken to extremes. The first author realised the enormity of the divide between French and British practices in 2001, when asked to write an endorsement for *The Silent Child: Exploring the World of Children Who Do Not Speak* by Laurent Danon-Boileau.^1^ The book's author was described as one of France's most respected child psychoanalysts, with a particular interest in language. The book contained a series of case studies of children who did not have any formal diagnoses but appeared to fit criteria for autism, intellectual disability or dyslexia. As Law^2^ noted in a review, there was no recognition of developments in social cognition and developmental psycholinguistics. Rather, there were confident assertions about the child's inner state, and children's lack of communication was generally attributed to affective and motivational causes relating to psychodynamic factors, rather than to any problems with understanding or formulating language.

To a child language expert, it is surprising that anyone should imagine psychoanalysis, the quintessential talking therapy, would help a child with impaired communication. Yet psychoanalysis is still taken seriously as an intervention for autism in a few countries, with France being the most notable example. This was documented in a film, *Le Mur*,^3^ produced in 2011 by journalist Sophie Robert. In a series of interviews, child psychoanalysts interpreted language limitations in their young clients through the lens of psychoanalytic theory. The parents were directly implicated in causing autism, and the child's communicative problems were regarded as a reflection of a difficult parent–child relationship. The flavour of the content is conveyed by a few direct quotes from the English transcript.

‘What we can notice when we take care of autistic children is precisely that autistic children are sick of language. That autism is a way to defend themselves from language.’ (Esthela Solano)

‘At the beginning, the child thinks that he is his mother’s phallus. Namely that he is this object that would give everything, fill his mother with joy, make her have orgasm … The father is there to forbid and at the same time protect the child. That is, to protect the child from the incestuous desire of the mother.’ (Yann Bogopolsky)

Laurent Danon-Boileau was one of the interviewees. It was illuminating to hear him describe a session with an autistic child; he did not seem to see his part as to facilitate the child's development, but rather to adopt a passive, interpretative role.

*Le Mur* was shocking for autism experts outside France. Although there are child psychoanalysts in the English-speaking world, they are a small minority and recommendations by the National Institute for Health and Care Excellence^4^ for intervention in autism make no mention of this approach. In the UK, autism is regarded as a neurodevelopmental condition with a predominantly genetic aetiology, and intervention focuses on working directly with social communication and behaviour. The goal is to bring about an improved developmental trajectory, rather than gaining any insights into the deeper meaning of the symptoms.

## French cultural and political influences on acceptance of psychoanalysis

It is worth reflecting on how such differences in practice have come about. Language barriers play a part in preventing the flow of ideas across national borders, but this cannot account for the unique status of psychoanalysis in France, where it is celebrated and protected.

Houzel,^5^ in recounting the history of psychoanalysis as a treatment for autism, noted that French society has ‘a very marked cultural identity that results in a certain impermeability to external currents of thought’ (p. 742). For Houzel, himself a psychoanalyst, this has certain benefits: ‘One advantage is a capacity to resist certain fashions that is no doubt greater than elsewhere. That is why France remains today a country where the application of psychoanalysis to the treatment of autists persists in spite of all the attacks that it is subjected to’ (p. 742).

Houzel further noted how French intellectuals are drawn to abstraction and speculative theorisations; these are exemplified by the work of Jacques Lacan, who did not himself work with children but developed an influential system of thought, based on Freudian theory, with its own specific French flavour. Its appeal to French intellectuals remains a mystery to those of us with a less credulous frame of mind, who suspect that when an author writes obscurely it is not because the ideas are brilliant, but because they are using verbiage to hide muddled thinking. As Billig^6^ concluded: ‘academic authors do not advance the cause of critique by putting their readers in a subservient position where authority has to be taken on trust and where obscurity takes priority over clarity’ (p. 22).

Houzel's paper put the development of psychoanalysis in France in a historical context and noted its cultural and political influences, but it also emphasised the extent to which psychoanalysis, as a treatment for autism and related developmental disorders, has come under repeated criticism over recent decades. Some parent organisations have attacked psychoanalysis on the basis that their children are denied the kind of interventions that are routinely available in other countries.^7^ In 2012, a political bill was put forward to the French National Assembly calling on the government to ‘condemn and prohibit psychoanalytic practices in all their forms concerning the treatment of autism’.^8^ In parallel with these developments, there have been a series of National Autism Plans, starting in 2005; there is now significant funding of research from the perspective of autism as a neurodevelopmental disorder and a cadre of French autism researchers with international standing.^9^ Yet French psychoanalysts remain politically powerful and have waged a campaign against those who challenge them, including an attempt to sue director Sophie Robert on the grounds that *Le Mur* misrepresented their views.^7^

## Psychoanalytic interpretations of maternal factors in aetiology

Houzel^5^ suggested that the chief reason for rejection of psychoanalysis is a mistaken belief that psychoanalysis blames mothers for their children's difficulties. He argued that this is not the case: psychoanalysis is not adequate for identifying aetiologies, and ‘Its quest is more in the direction of meaning than that of cause’ (p. 731).

To maintain that psychoanalysis does not blame parents seems disingenuous. There are many varieties of psychoanalysts, and it is true that some, including most British psychoanalysts, overtly state that parents should not be blamed for their child's difficulties.^10^ Nevertheless, in France, the role of parents, especially mothers, in causing disorders has been a core feature of psychoanalytic work with children. Briggs^11^ noted how the work of Bruno Bettelheim had been influential in France, with his view of cold, rejecting mothers (the classic ‘refrigerator mother’ of Kanner) from whom the child withdraws into his shell. Bates^7^ cited numerous examples of mothers being told bluntly that they were responsible for their child's autism, although the more common accusation was that mothers were overinvolved and ‘smothering’, with an unhealthy desire for the child that led to the child being unable to achieve a separate identity. *Le Mur*, and a more recent film by Sophie Robert, *Le Phallus et le Néant*,^12^ contain several examples of psychoanalysts putting forward such viewpoints.

Bates^7^ noted that these ideas fell on fertile soil in France, as they fitted well with notions of toxic mothers already endorsed by French psychoanalysts. Several relevant quotes by Françoise Dolto, one of the most influential French child psychoanalysts of the last century, can be found on the Freud Quotidien website,^13^ including this on autism:

‘The child wants what they see the adult wanting. If they focus the desire of the adult, the source of the desire in them dries up and what remains of it is inflected on their own vegetative material person, causing autism, that is to say disorders of its spatio-temporal reference and of communication. This mental illness, leading in the worst case to infantile dementia in a previously open and intelligent baby, is established in the infant separated from all their references. It has also been called “hospitalism” which, at all levels, depending on the duration of the pain, is in fact a disease of the desire. While needs are preserved, desire loses in this child its magnetic vector calling for communication. but “hospitalism” can also be observed in a family environment, in infants whom the mother or rather the neurotic employee isolates in an obsessive way by exclusive possessiveness, or who is the object of perfect care, technically speaking, given without joy by a depressive adult’ (translated by Julien Basch).

## Rejection of the need for a conventional evidence base for psychoanalysis

Houzel also bypassed two further objections to psychoanalysis, which are particularly concerning to any dispassionate observer of French child psychiatry. The first is the lack of any accepted evidence base for psychoanalytic treatments. Houzel^5^ regarded behavioural approaches to intervention as mere fashions in reductionist thinking, and noted that they have not been strikingly successful in gaining understanding of the nature or causes of autism, nor in creating improved outcomes for children. This point has some justification – progress has certainly been slow and there is no miracle cure.^14^ The difference compared with psychoanalysis, however, is that these developments occur within a scientific framework that allows one to test the ideas and reject those where the evidence does not fit. Popper^15^ used psychoanalysis as one of his classic examples of pseudoscience, able to explain all phenomena but with no possibility of being disproven: if the scientific framework is itself rejected, then any viewpoint is as valid as another. Billig^6^ pointed out that Lacan's supporters ignored attempts by experimental psychologists to evaluate his work, because they regarded orthodox psychology as invalid; yet, even when considered in its own terms, Lacan's citation of evidence was sloppy and inaccurate. Sokal and Bricmont^16^ were particularly harsh on Lacan, for producing obscure writings with all the trappings of technical language and concepts but no coherent meaning. Consistent with this, Law^2^ noted that Danon-Boileau^1^ did not engage in any discussion of evidence-based practice and indeed seemed to make a virtue of his lack of reading in the area. The impression is that many French psychoanalysts regard themselves as revolutionary thinkers who, in rejecting mainstream science, are challenging the conventional power structures in society. But they ignore the potential for abuse of adults’ power over children, who are defenceless against having their thoughts and motivations interpreted in terms of the analyst's unevidenced theory.

## Psychoanalytic accounts of sexual relations between adults and children

A different type of critique of French psychoanalysis is highlighted in Robert's most recent film, namely, that it has been used to validate incest and child abuse. Freud, and his follower Lacan, regarded children as sexual beings, strongly influenced by erotic desire for a parent and preoccupied by concerns about castration, lack of a penis or violence. Given that these are seen as universal human desires, incest and paedophilia are regarded as natural phenomena. According to this view, psychic conflicts are largely due to the need to fit in with the norms of a society that strictly prohibits such behaviours and hence to repress natural instincts. The focus on child sexuality was one reason that many of Freud's contemporaries ultimately broke away from him;^17^ he was seen as imposing his own dogmatic views, derived from his self-analysis, on others, treating concepts such as the Oedipus complex and castration anxiety as universal, to the neglect of other, non-sexual risk factors for mental disturbances. In *Le Phallus et le Néant*, we see how this viewpoint can open the way for abusive relationships between a powerful therapist and vulnerable children.

The risk of abuse needs to be viewed in relation to a distinct French cultural perspective with regard to an age of consent.^18^ In 1977, a group of 60 prominent intellectuals signed a petition that was published in *Le Monde*, coinciding with the trial of three men who had been accused of having sex with 13- to 14-year-old children. The argument in the petition was that children had the capacity to consent to sex, and that adoption of an age of consent was patriarchal and a denial of children's rights. A similar petition was published 1979, in support of a man on trial for having sex with girls between the ages of six and 12. ‘Desire and sexual games have their place in the relationship between children and adults’ was the argument put forward, with the claim reiterated that children's rights were being abused by denying them sexual gratification.

One signatory of the 1977 petition was Françoise Dolto, a media-friendly psychoanalyst who regularly appeared on a radio programme between 1976 and 1978, where she answered parents’ questions. In 1979 she was interviewed by the magazine *Choisir* on the topic of incest; a transcript can be found online.^19^ Her responses indicated that she regarded children as willing participants in sexual activity who should take responsibility for their actions. The same website reported quotes from a 1999 book, *L'Enfant, le Juge et la Psychanalyste*, in which Dolto discussed with judge Andrée Ruffo the legal implications of sexual relationships between adults and children. Dolto recommended that children should be taught early that sexual contact with an adult is against the law, so that there will be no doubt about their complicity if they do engage in such activities:

‘If children knew that the law prohibited sensual privacy between adults and children, well, from the moment an adult asks her, if she accepts, that makes her an accomplice, she doesn't have anything to complain about’ (authors’ translation).

Dolto continues to be held in high regard in France. Not only has Paris named a street after her, but in 2018 the government printed a special postal stamp in her honour. *Le Phallus et le Néant* makes it clear to what extent her legacy lives on, with interviewees maintaining that children are sexual beings who are capable of behaving provocatively towards their parents, who must repress their ‘inner paedophile drive’.

Such discourse is not merely symbolic: it has real consequences for children. Growing unease in French society about the cultish status of psychoanalysis came to a head this year with the publication of a book *Le Consentement* by Vanessa Springora,^20^ who described how at the age of 14 she was drawn into a sexual relationship with a celebrated 50-year-old writer, Gabriel Matzneff. Matzneff was one of the signatories of the 1977 petition for decriminalising paedophilia. He defended himself by arguing that his relationship with the teenaged Springora and others of her age were love affairs.

No doubt there are many child psychoanalysts who would be horrified at the notion that their methods were being used to defend incest and child abuse. The problem, though, is that if someone were inclined towards paedophilia, then Dolto's version of psychoanalysis would appear very attractive, promoting as it does the idea that sexual relationships between adults and children, while prohibited by society, are a natural and therefore blameless aspect of the human condition. Psychoanalysis can provide professional respectability, a good income and access to vulnerable children. We should be clear: we are not saying that these views are common among French child psychoanalysts. Nevertheless, so long as the psychoanalytic movement in France sets no limits as to what can count as psychoanalysis, it runs the risk of causing harm to children, as well as to its profession.

## Continuing power of psychoanalysis in French political and academic life

The key question is no longer how France arrived at this point but rather how it cannot seem to fully get beyond it. Although psychoanalysis is now marginal in France for psychiatry as a whole, it is a different story for the subdiscipline of child psychiatry that has been dominated by this orientation for decades. In 2012, the High Health Authority of France implemented recommendations for the treatment of autism, but they were not obligatory and inefficient psychoanalytical therapies continued to be proposed for individuals with autism.^21^ Even though new generations of physicians are trained in evidence-based treatments, the older generations that were trained to see psychoanalysis as a viable treatment for autism are still in practice. This presence is visible at all levels of the French healthcare system, including public hospitals, clinics and private practice. Perhaps the biggest problem in France concerns the training of clinical psychologists. Psychologists are ten times more numerous than psychiatrists, and they occupy a large number of positions in clinics and hospitals treating children with autism. The second author, an expert for the National University Council (Conseil National des Universités), recently provided a scientific criticism of psychoanalysis as well as quantitative analysis of the training received by clinical psychologists in French universities.^22^ This analysis demonstrated that of the 26 universities charged with the training of clinical psychologists, half still provide substantial psychoanalytic training. In nine of these universities, the training provided in clinical psychology is *exclusively* psychoanalytic in orientation. Clinicians trained in these institutions are not routinely exposed to evidence-based approaches in the treatment of autism (or other mental disorders, for that matter), and no national examinations or professional licensing criteria require them to have such training before assuming positions at hospitals throughout the country. The French government and university presidents have turned a blind eye to this psychoanalytic monopoly at institutions of higher education.

In sum, the defence of psychoanalysis as a treatment for autism rests on the idea that choice of one form of therapy over another is purely due to cultural preferences and fashion. A deeper investigation, however, reveals that psychoanalysis is qualitatively different from other forms of therapy. It is not only bereft of any evidence of effectiveness, but it is so ill-defined that it is unclear what such evidence would look like. It is only legitimised because it is promoted by authority figures and maintained by circles of power and influence. Moreover, in its more extreme forms, it has potential to cause damage to parents, especially mothers, who are demonised both for being too involved with and too remote from their children, and to children themselves, who are regarded as seducers rather than victims when involved in sexual relationships with adults.

## About the authors

Dorothy Bishop, M.A., M.Phil., D.Phil., is Professor of Developmental Neuropsychology in the Department of Psychology, University of Oxford, UK. Joel Swendsen, M.A., Ph.D., is Director of Research at the National Centre for Scientific Research, France.
